# Generate the scale-free brain music from BOLD signals

**DOI:** 10.1097/MD.0000000000009628

**Published:** 2018-01-12

**Authors:** Jing Lu, Sijia Guo, Mingming Chen, Weixia Wang, Hua Yang, Daqing Guo, Dezhong Yao

**Affiliations:** a The Clinical Hospital of Chengdu Brain Science Institute, MOE Key Lab for Neuroinformation; b School of Life Science and Technology, Center for Information in BioMedicine, University of Electronic Science and Technology of China; c Department of Composition, Sichuan Conservatory of Music, Chengdu, Sichuan, China.

**Keywords:** BOLD, brain music, DTI, neural mass model, scale free

## Abstract

Many methods have been developed to translate a human electroencephalogram (EEG) into music. In addition to EEG, functional magnetic resonance imaging (fMRI) is another method used to study the brain and can reflect physiological processes. In 2012, we established a method to use simultaneously recorded fMRI and EEG signals to produce EEG-fMRI music, which represents a step toward scale-free brain music. In this study, we used a neural mass model, the Jansen–Rit model, to simulate activity in several cortical brain regions. The interactions between different brain regions were represented by the average normalized diffusion tensor imaging (DTI) structural connectivity with a coupling coefficient that modulated the coupling strength. Seventy-eight brain regions were adopted from the Automated Anatomical Labeling (AAL) template. Furthermore, we used the Balloon–Windkessel hemodynamic model to transform neural activity into a blood-oxygen-level dependent (BOLD) signal. Because the fMRI BOLD signal changes slowly, we used a sampling rate of 250 Hz to produce the temporal series for music generation. Then, the BOLD music was generated for each region using these simulated BOLD signals. Because the BOLD signal is scale free, these music pieces were also scale free, which is similar to classic music. Here, to simulate the case of an epileptic patient, we changed the parameter that determined the amplitude of the excitatory postsynaptic potential (EPSP) in the neural mass model. Finally, we obtained BOLD music for healthy and epileptic patients. The differences in levels of arousal between the 2 pieces of music may provide a potential tool for discriminating the different populations if the differences can be confirmed by more real data.

## 1 Introduction

Music has existed in human society since prehistory.^[1]^ Due to its long history, music is considered an artistic expression and can represent the human mind or mood. Additionally, music can shape our brain through long-term training.^[2,3]^ How do our brains process music into something emotionally powerful, and how does music affect us? The sonification of brain signals is an approach to study the relationship between the brain and music.^[4]^ In addition, this approach could facilitate studies of brain mechanisms. Since 2009, we have been developing methods to translate electroencephalogram (EEG) signals, 1 form of neuroinformation, into music. To date, several methods have been developed to generate scale-free music according to the varieties of neuroinformation.^[5,6]^ Thus, we can understand the meaning of brain rhythms from a musical perspective and examine the physiological mechanism behind the neuroactivities.

Currently, diffusion tensor imaging (DTI), which enables visualization and characterization of white matter fasciculi, has become one of the most popular magnetic resonance imaging techniques in brain research. Information transmission in the brain is assumed to be mediated by the underlying brain structure, and the fiber tracts have been used to reflect the anatomical connectivity of the brain.^[7,8]^ In several recent studies, various mathematical models have been developed using the DTI structural connection matrix to simulate both normal and abnormal brain states; thus enabling a better understanding of different brain functions.^[9–11]^

Epilepsy is a common nervous system disease worldwide. Epilepsy is a chronic disease caused by brain neurons suddenly becoming abnormal, leading to transient brain dysfunction; epilepsy plagues the physical and mental health of humans. EEG is a very important tool for processing epilepsy diagnosis, and it can reflect the special waveforms generated by epilepsy during seizures. However, the accuracy of clinical diagnosis by EEG is not very high.^[12]^ Furthermore, because epilepsy is unpredictable, it is difficult to obtain the real-time signal.

The neural mass model, which is a classical macroscopic population model, has been used to study brain signals for many years. Additionally, this model has greatly advanced our understanding of brain functions.^[13–22]^ The Jansen–Rit neural mass model is widely used to study the brain's various EEG rhythms.^[23–25]^ Compared to real physiological data, the data produced by the model are in line with physiological characteristics. More importantly, the physiological parameters generated by the model can be used to predict the neural mechanisms concealed under real physiological data.^[26]^ The blood-oxygen-level dependent (BOLD) signal is one of the physiological signals that can reflect neural activities. However, the temporal resolution of the BOLD signal is too low to generate music for further analysis. To address this issue, we simulated BOLD activity in each cortical region based on the Jansen–Rit model and the Balloon–Windkessel hemodynamic model, and then, we obtained the signal with a higher temporal resolution.

Here, we are interested in using BOLD music, which was simulated by a whole-cortical brain network model constructed from DTI, to determine the relationship between the structure and function of the brain and music. Then, we translated this brain activity into scale-free brain music using the method described in our previous work.^[5,6]^ Unlike direct brainwave music, music generated from a neural mass model might reflect various physiological brain states and enable us to develop another tool for understanding brain disease, such as epilepsy.

## 2 Materials and methods

### 2.1 Data acquisition

DTI data were obtained from 15 healthy volunteer students from the University of Electronic Science and Technology of China (UESTC) on a 3T MRI scanner (GE Discovery MR750) at the MRI research center of UESTC. Our research was approved by the Ethics Research Committee at the School of Life Science and Technology, UESTC. All subjects had no history of clinical evidence of major neurological or psychological disorders. The subjects provided informed consent before the experiment was conducted according to the established guidelines of the review boards and were paid for their participation.

As previously described,^[27]^ each diffusion-weighted imaging dataset consisted of a nondiffusion weighted volume with *b* = 0 and 20 diffusion weighted images with the following parameters: 50 slices of 2.5-mm thickness, with 3.25 mm between adjacent slices; *b* = 1000 s/mm^2^ for the weighted images; field of view = 220 × 220 mm^2^; acquisition matrix = 128 × 128, corresponding to an “in plane” spatial resolution of 1.72 × 1.72 mm^2^; echo time/repetition time = 104 ms/7200 ms; and a flip angle = 90°. For the 3D T1-weighted images, we used the following scan parameters: 176 contiguous slices of 1-mm thickness in the sagittal orientation; in plane field of view = 224 × 256 mm^2^, with a spatial resolution of 1 × 1 mm^2^; echo time/repetition time = 3.02 ms/2600 ms; and a flip angle = 8°.

**Figure 1:**
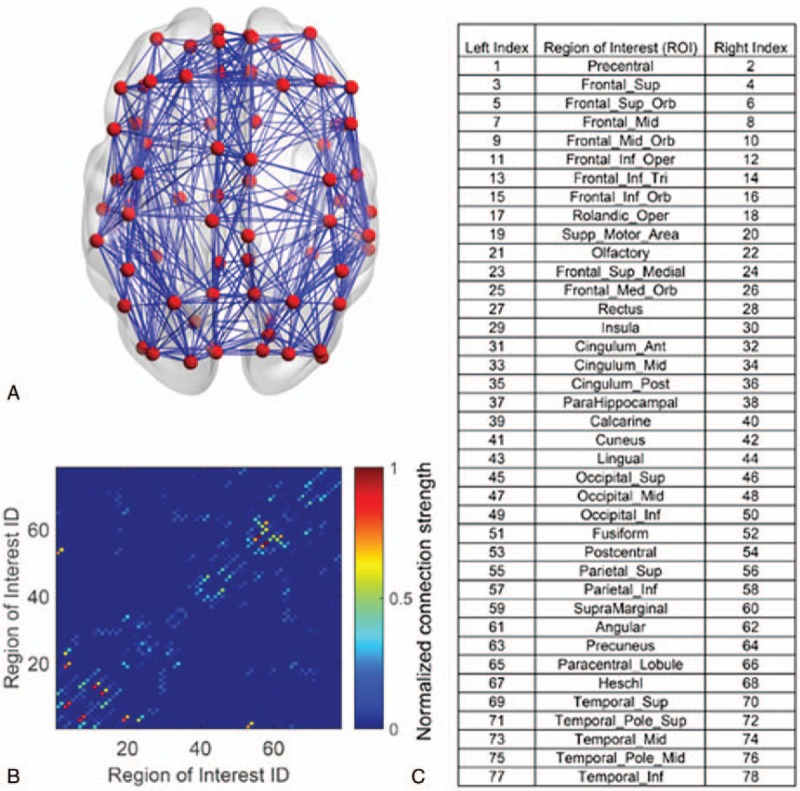
Anatomical structure of human subjects. (A) The averaged structural connectivity network from 15 healthy human subjects. The red nodes indicate the cortical region of interest (ROI), and the blue lines show the fiber connectivity between two cortical regions. (B) The averaged structural connectivity matrix. Note that there are 78 cortical ROIs, and the connectivity strength is normalized with the maximal connectivity strength in the matrix. (C) The list of cortical regions is based on the AAL template. Here, the left and right indices show the cortical regions in the left and right hemispheres, respectively. AAL = Automated Anatomical Labeling, ROI = region of interest.

To map the connections between brain regions, we used FSL software (FSL4.1.6, http://www.fmrib.ox.ac.uk/fsl) to process and analyze the data. The diffusion parameters were estimated to build up the distributions of the diffusion parameters at each voxel. Then, probabilistic tracking was performed on each mask. Finally, the connectivity matrix (shown Fig. 1A and B) was established by taking the sum of the connection weight from region X to region Y and that from region Y to region X as the weight of the edge between regions X and Y.^[27]^ We used the connectivity matrix as the basis of the model structural connection to mediate the interactions between different cortical regions. Each element in the connectivity matrix represents the normalized fiber connection strength between related brain regions. It should be noted that in this study, we adopted only 78 cortical regions based on the Automated Anatomical Labeling (AAL) template.^[28]^ The corresponding brain regions are shown in Fig. 1C.

### 2.2 Jansen–Rit neural mass model

**Figure 2:**
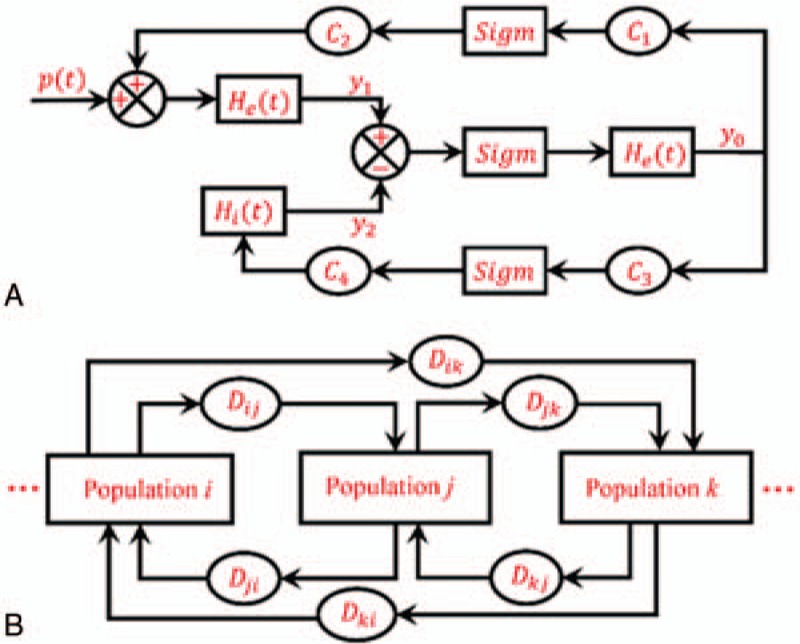
Schematic of the neural mass model. (A) The framework of the Jansen–Rit model, which contains a pyramidal neuron population and excitatory and inhibitory interneuron populations. (B) The cortical network model based on the Jansen–Rit model. *D*_*i,j*_ shows the connection strength from cortical region *i* to *j*.

In the present study, we used the Jansen–Rit model,^[15]^ which is a classical neural mass model derived from the lumped parameter model,^[13]^ to simulate the activity of each cortical brain region. As previous studies described, the neural mass model consists of 3 neural populations: a pyramidal neuron population, excitatory interneuron population, and inhibitory interneuron population. Each population connects to the other populations with numerous synapses characterized by connectivity constants (see Table 1). The simplified schematic of each cortical model is shown in Fig. 2A.

**Table 1:**
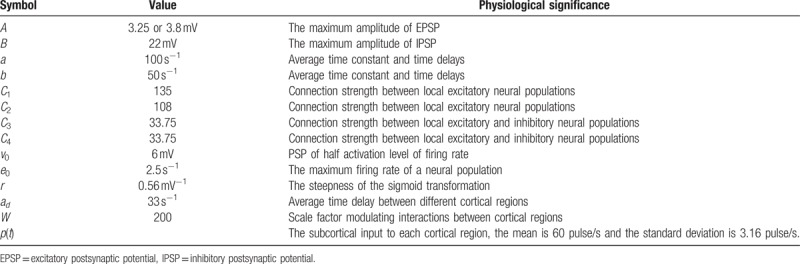
The parameters used in the model are adopted from previous studies.

In each neural population, the transformation relationship between the 2 main variables, which are the average postsynaptic potential and pulse density of the action potential, determines the dynamical behavior of the neural population. To clearly show the relationship, we used a linear block and a nonlinear block. Specifically, the linear block transformed the average pulse density of action potentials from other populations into the average postsynaptic membrane potential. Following Jansen and Rit,^[15]^ the impulse response was different for excitatory and inhibitory neural populations, which is shown as 
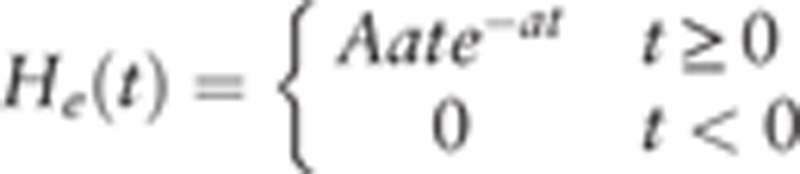
 and 
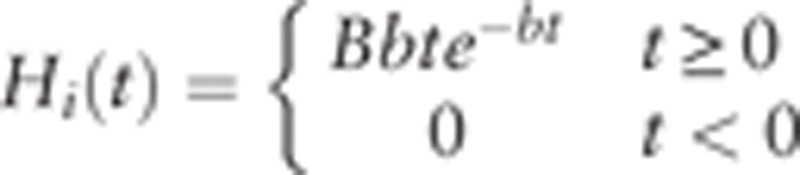
, respectively. *A* and *B* depict the amplitude of the excitatory and inhibitory postsynaptic potentials (EPSP and IPSP), respectively, *a* and *b* are the lumped parameters of the reciprocal of the time constants for the EPSP and IPSP, respectively. Therefore, based on the impulse response function, we could infer that the form between the input signal and output signal matches a second order differential equation as 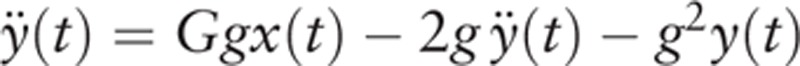, where *x*(*t*) and *y*(*t*) are the input and output signals, respectively. The value represented by *G*(*g*) was the same as those represented by *A*(*a*) and *B*(*b*).

The nonlinear block uses a sigmoid function to transform the average postsynaptic membrane potential into the average pulse density, which is shown as 
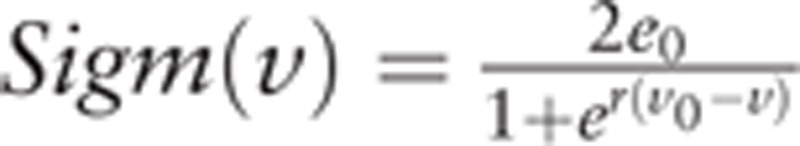
. Here, *e*_0_ indicates the maximum firing rate for each neural population, *r* is the steepness, and *v*_0_ is the half-activation level. Using this sigmoid function can ensure that the firing rate of each neural population is within a reasonable physiological range as the potential changes.

According to the 2 blocks for each neural population, the interactions between different neural populations can be clearly described by 6 ordinary differential equations, as described in previous work.^[15]^ Note that in the normal brain state, we set *A* = 3.25 mV; however, *A* = 3.8 mV for the epileptic case.^[15–17]^ The model parameters and related physiological significances are given in Table 2. All simulations were based on these default parameters unless otherwise stated.

**Table 2:**
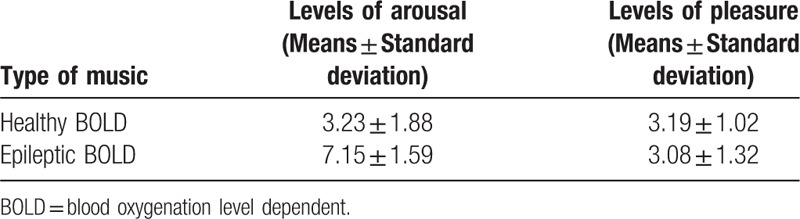
The levels of arousal and the levels of pleasure on BOLD music judged by 25 volunteers.

To show the brain interactions across large-scale cortical regions, we used the Jansen–Rit model to build a whole-brain cortical network model (Fig. 2B) with the averaged DTI connectivity matrix. Due to time delays caused by spatial factors of brain cortical regions, a time delay impulse response function was introduced. Following Jansen and Rit,^[15]^ the form of the delayed impulse function is defined as 
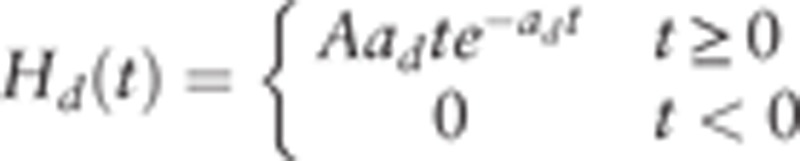
, which is similar to the excitatory impulse response function, and 
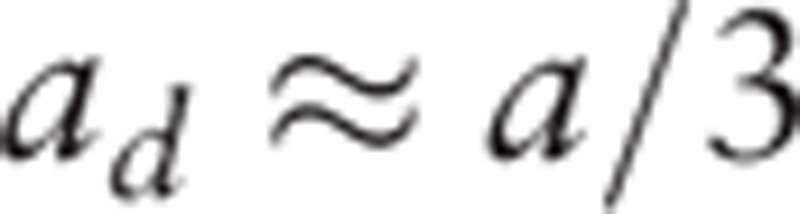
. Furthermore, we assumed that these impulse functions are the same for each brain cortical region. Hence, the large-scale cortical network model^[15,16]^ can be shown as 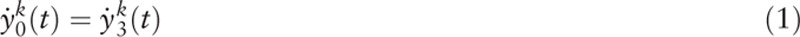 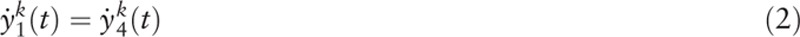 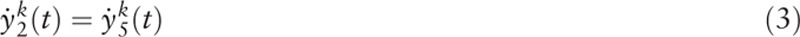 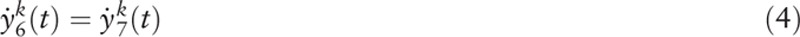 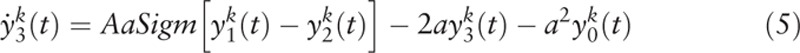 
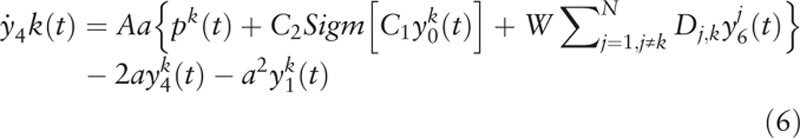
 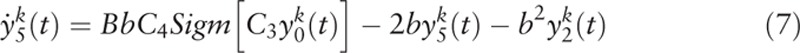 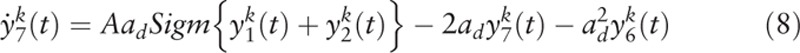


Here, *D*_*jk*_ shows the fiber connection strength from the cortical region *j* to *k*. *W* is the scale factor modulating the interactions between cortical regions, and we set *W* = 200 in our simulations. The number of cortical regions considered in the network model was *N* = 78. 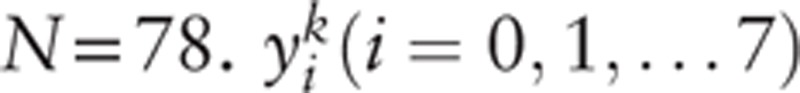 is the output of the excitatory or inhibitory neural population, and 
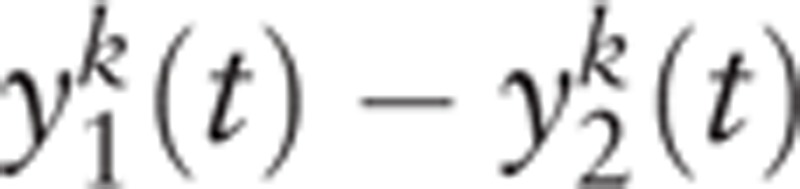
 is the output of the neural population in the cortical region *k*. In the following, we used the Balloon–Windkessel hemodynamic model^[29]^ to transform the neural population output 
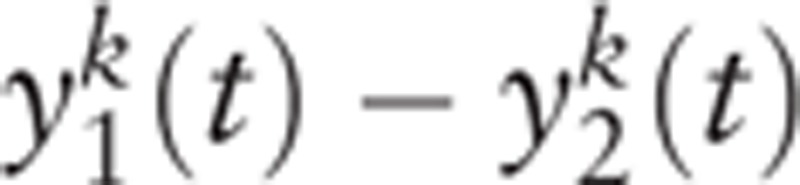
 into the BOLD signal. Note that in our simulations, we down-sampled the BOLD signal to 250 Hz to enable a comparison analysis.

All of the above differential equations in this cortical network model were solved numerically with a forward Euler method with a step size of 0.1 ms.

### 2.3 Brain music from the BOLD signals

**Figure 3:**
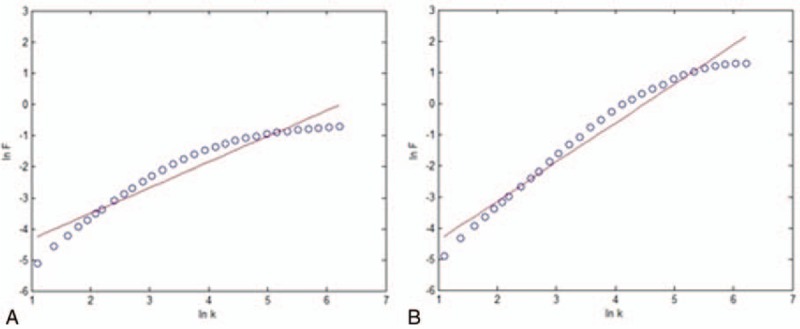
The power law for the generated BOLD signals. The logarithm of *F*(*k*) is plotted as a function of the logarithm of the time scale *k*.^[5]^ The slope of the plot, *α* = ln *F*/ln *k* (the scaling exponent α is specified for Fig. 3), is called the scaling exponent. The scaling exponent *α* of the healthy BOLD signal is 0.822, and the scaling exponent *α* of the epilepsy BOLD signal is 1.255, both of which obey the power law rule. BOLD = blood oxygenation level dependent.

**Figure 4:**
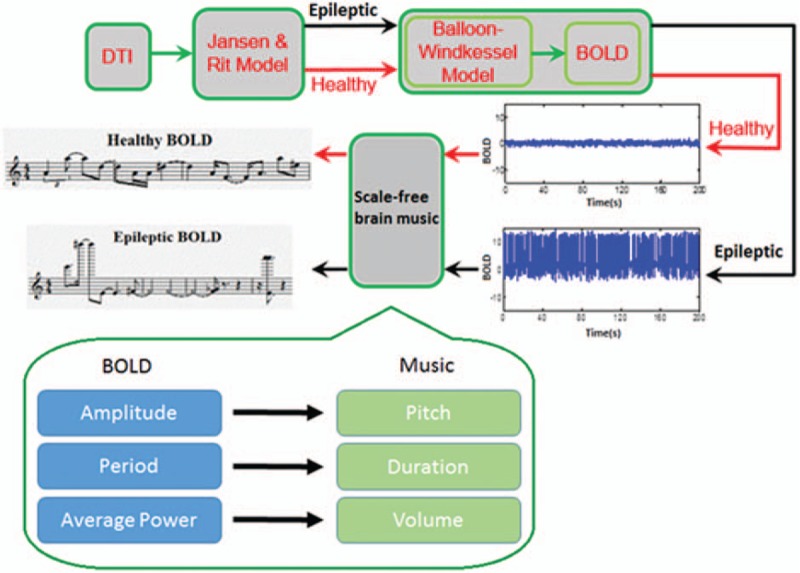
The procedure for generating the BOLD music. BOLD = blood oxygenation level dependent.

After the calculation, BOLD signals were found to obey power-law rules (shown in Fig. 3). We simulated the case of an epileptic patient by changing the parameter that determined the amplitude of the EPSP in the neural mass model. Therefore, we obtained 2 BOLD signals. Afterward, we chose a temporal region channel to generate the scale-free music according to the translation rules. The rules included the direct mapping from the BOLD signal period to the duration of a note, the logarithmic mapping of the average power change of the BOLD signal to the music intensity according to Fechner's law, and a scale-free-based mapping from the amplitude of the BOLD signal to music pitch according to the power law. The entire procedure from DTI to BOLD music is shown in Fig. 4.

## 3 Results

We obtained different BOLD music for healthy and epileptic cases (shown in Fig. 4). To test the distinct BOLD music between these 2 cases, we recruited 25 volunteer students from UESTC to judge differences between these pieces of music. None of the volunteers had professional training in music. The volunteers were asked to rate arousal level (1-weak, 9-strong) and the pleasure level (1-weak, 9-strong), which are commonly used in brief evaluations of music.^[30]^ The results are shown in Table 2. The significant musical distinction on levels of arousal (*t* = 8.11, *P* < .05) provides a potential tool for discriminating different populations if these differences can be confirmed by real data. However, there was no significant difference in levels of pleasure (*t* = 0.35, *P* > 0.05) between these 2 types of music.

## 4 Discussion

The brain music, containing individual physiological information, is derived from physiological signals.^[31]^ Thus, the potential clinical application makes it intriguing. Throughout human physiological signals, BOLD may relate to the electrical activity of a group of neurons through a neurovascular coupling relationship. However, the sonification of BOLD signals has rarely been reported, which is probably due to its low temporal resolution. Based on the Jansen–Rit model and the Balloon–Windkessel hemodynamic model, we can obtain the BOLD signal with a higher temporal resolution and make its sonification feasible.

Furthermore, the different scaling exponent *α* based on detrended fluctuation analysis (DFA) has different meanings.^[32]^ Some studies have shown that DFA has a good effect on the clinical detection and classification of epilepsy.^[33,34]^ A study on DFA exponents for seizure activity in human hippocampus found a small enhancement of α during seizure activities.^[35]^ In our epileptic case, the scaling exponent *α* of the BOLD signal from the healthy and the epilepsy is different, which is in accordance with previous research. In addition, differences in the levels of arousal of BOLD music between healthy and epileptic music are also found. We believe BOLD music can be a potential tool for discriminating different populations.

Nonetheless, it should be noted that the model we used in this article was not perfect. In our study, we only adopted 78 cortical regions based on the AAL template; this division for the use of models may not be accurate enough. In addition, the individual differences should be considered in the model in the following study. Future work may also focus on whether BOLD music associated with these musical differences can provide more potential applications after being confirmed by real data.
